# Odd chain fatty acid metabolism in mice after a high fat diet

**DOI:** 10.1016/j.biocel.2021.106135

**Published:** 2022-02

**Authors:** Isaac Ampong, O. John Ikwuobe, James E.P. Brown, Clifford J. Bailey, Dan Gao, Jorge Gutierrez-Merino, Helen R. Griffiths

**Affiliations:** a Faculty of Health and Medical Sciences, University of Surrey, GU2 7XH, UK; b School of Life and Health Sciences, Aston University, Birmingham B4 7ET, UK; c Xi’an Jiaotong University Health Science Center, Xi’an, China

**Keywords:** Free fatty acids, Gut microbiota, Odd-chain saturated fatty acids, Steatosis, Liver

## Abstract

Epidemiological studies show that higher circulating levels of odd chain saturated fatty acids (FA: C15:0 and C17:0) are associated with lower risk of metabolic disease. These odd chain saturated fatty acids (OCSFA) are produced by α-oxidation in peroxisomes, de novo lipogenesis, from the diet and by gut microbiota. Although present at low concentrations, they are of interest as potential targets to reduce metabolic disease risk. To determine whether OCSFA are affected by obesogenic diets, we have investigated whether high dietary fat intake affects the frequency of OCSFA-producing gut microbiota, liver lipid metabolism genes and circulating OCSFA. FA concentrations were determined in liver and serum from pathogen-free SPF C57BL/6 J mice fed either standard chow or a high fat diet (HFD; 60% calories as fat) for four and twelve weeks. Post-mortem mouse livers were analysed histologically for fat deposition by gas chromatography–mass spectrometry for FA composition and by qPCR for the lipid metabolic genes fatty acid desaturase 2 (*FADS2*), stearoyl CoA desaturase 1 (*SCD1*), elongation of long-chain fatty acids family member 6 (*ELOVL6*) and 2-hydroxyacyl-CoA lyase 1 (*HACL*). Gut microbiota in faecal pellets from the ileum were analysed by 16S RNA sequencing. A significant depletion of serum and liver C15:0 (>50%; P < 0.05) and liver C17:0 (>35%; P < 0.05) was observed in HFD-fed SPF mice in parallel with hepatic fat accumulation after four weeks. In addition, liver gene expression (*HACL1, ELOVL6, SCD1* and *FADS2*) was lower (>50%; P < 0.05) and the relative abundance of beneficial C3:0-producing gut bacteria such as *Akkermansia*, *Lactobacillus*, *Bifidobacterium* was lower after HFD in SPF mice. In summary, high dietary fat intake reduces serum and liver OCSFA, OCSFA-producing gut microbiota and is associated with impaired liver lipid metabolism. Further studies are required to identify whether there is any beneficial effect of OCSFA and C3:0-producing gut bacteria to counter metabolic disease.

## Highlights

• Liver and microbiota metabolism are known to affect odd chain fatty acid (OCFA)levels.
• A high fat diet (HFD) lowered plasma C15:0 and liver C15:0 and C17:0 in mice.
• HFD-fed mice expressed lower *HACL1*, *ELOVL6*, *SCD1* and *FADS2*, and accumulated liver fat.
• Relative abundance of *Akkermansia*, *Lactobacillus*, *Bifidobacterium* was lower after HFD.
• HFD reduced OCFA levels, OCFA-producing gut microbiota and hepatic FA metabolism.

## Graphical Abstract

**Figure:**
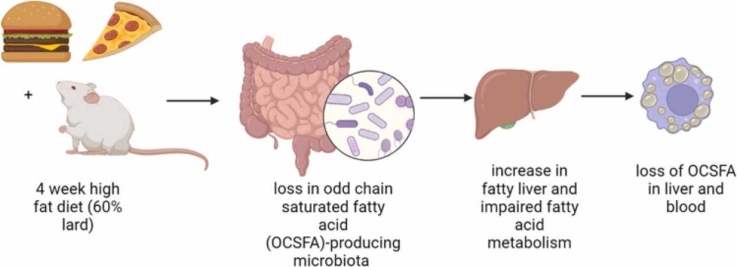

## 1 Introduction

Obesity is a significant risk factor for metabolic disease development (Ravussin and Smith, 2002). It is strongly influenced by lifestyle factors such as exercise, diet (Ravussin and Smith, 2002, Riccardi et al., 2004), and by the nature of the dietary fatty acids (FA) that are consumed. The accumulated fat is stored within rapidly expanded visceral tissue depots (von Frankenberg et al., 2017).

Similarly, ageing is associated with redistribution of fat away from subcutaneous depots into visceral tissues and saturated fatty acids (SFAs) accumulate within these ectopic fat stores, (Conte et al., 2019). Since the rate of efflux of FAs acids into the circulation is higher from visceral compared to subcutaneous stores (Gentile et al., 2015), the higher levels of SFA in plasma from older adults (Pararasa et al., 2016) and in obese people may be explained at least in part by increased fat storage in visceral tissues. A pathophysiological increase of circulating total SFA contributes to insulin-resistance and increases the risk of developing type 2 diabetes mellitus (T2DM) (Riccardi et al., 2004). Consistent with clinical observations, many in vitro studies have shown that elevated concentrations of some SFA, particularly palmitate (C16:0) are toxic to a variety of cells including macrophages, adipocytes, muscle, islet cells and hepatocytes (Gao et al., 2012, Gao et al., 2009, Zhang et al., 2020, Den Hartogh et al., 2020, Nakamura et al., 2009) by common mechanisms, e.g. uncoupling mitochondrial respiration and increasing production of reactive oxygen species (Nakamura et al., 2009, Borradaile et al., 2006, Deguil et al., 2011).

In contrast to the increased risk for metabolic disease that is described in people with elevated total SFA levels, a lower risk for T2DM has been associated with higher red cell membrane proportion of the odd chain saturated fatty acids (OCSFA), pentadecanoic acid (C15:0) and heptadecanoic acid (C17:0) within phospholipids (Krachler et al., 2008, Forouhi et al., 2014). In a prospective case-control study, a positive correlation between even chain SFAs (C14:0, C16:0, and C18:0) and T2DM has been observed, however, OCSFA (C15:0 and C17:0) were inversely associated with T2DM (Forouhi et al., 2014). Similarly, the serum levels of phospholipid C15:0 and C17:0 correlated inversely with non-alcoholic fatty liver disease (NAFLD) and hepatocyte ballooning scores (Yoo et al., 2017). A recent analysis of the EPIC cohort confirmed that total C15:0 and C17:0 are the strongest metabolic signatures of healthy lifestyle pattern and lower colon cancer risk (Venn-Watson et al., 2020). These observations from prospective and epidemiological studies highlight that the biological activity of SFAs is not homogeneous.

OCSFA are present in lower abundance than SFA such as C16:0 in tissues and in the blood. They arise principally from peroxisomal α-oxidation, de novo lipogenesis in the liver and adipose tissue (including from OCSFA, released into the hepatic portal vein from gut-derived microbial metabolism), and directly from the diet (Jenkins et al., 2017). Hence, circulating C15:0 and C17:0 levels are influenced by different factors; in one study, total C15:0 levels correlated directly with dietary intake, whereas C17:0 was substantially synthesised by α-oxidation (Jenkins et al., 2017). However, another study in humans described that both C15:0 and C17:0 levels in blood were increased following dietary supplementation with either fermentable inulin or with C3:0, implicating intestinal bacteria involved in the fermentation of fibre as the relevant source of both circulating phospholipid OCSFAs (Weitkunat et al., 2017). A further study in rodents showed that an obesogenic HFD supplemented with C3:0 directly led to increased phospholipid C15:0 and C17:0, and that C15:0 levels were correlated with attenuation of HF-diet induced insulin resistance, likely due to effects on hepatic metabolism (Weitkunat et al., 2016). However, we have little insight into factors influencing OCSFA levels after an obesogenic HFD. Therefore, we investigated the hypothesis that an obesogenic diet reduces circulating OCSFA, the frequency of OCSFA-producing gut microbiota and impairs hepatic FA metabolism.

## 2 Materials and methods

### 2.1 High dietary fat study in mice

Four-week old C57BL/6 J male mice were maintained in Nanjing- China SPF animal facility on a 07.00–19.00 h day/light cycle at 20–22 °C with food and water ad libitum. They were housed 2–4 per cage and randomised to one of two diets - either control diet (CD; 10% fat, 70% carbohydrate and 20% protein per kcal%) or high fat diet (HFD; a 60% fat (as lard), 20% carbohydrate and 20% protein per kcal%) ad libitum for 4 weeks. The fatty acid composition of each diet is included in Table 1.

**Table 1:** Fat and fatty acid composition of diets.

Fat (g)	Chow control Diet	High Fat Diet
Lard	20	245
Soya bean fat	25	25
Total Fat	45	270
**Fatty Acid (g/kg diet)**		
14:0	0.3	2.8
15:0	0	0.2
16:0	6.4	49.9
16:1	0.3	3.4
17:0	0.1	0.9
18:0	3.1	26.9
18:1n9	12.3	86.3
18:2n6	17.8	72.7
18:3n3	2.1	5.1

All the procedures were approved by the Animal Use and Care Committee of the School of Life Science and Technology, Xi’an Jiaotong University. There were no adverse events. The body weight of the mice (n = 10/group) was taken weekly. At the end of the study, livers were removed, and the tissues were snap frozen for mRNA analysis for FA analysis and stored at − 80 °C or fixed in 4% formaldehyde for histological analyses. Serum was snap frozen and faecal samples were also stored at − 80 °C until analysis. The study was replicated in mice maintained in normal husbandry conditions over 4 and 12 weeks to understand the effect of dietary feeding time and of pathogen-free conditions.

## 3 Fatty acid (FA) analysis by GC-MS

FA extraction from serum was performed as previously described (Pararasa et al., 2016). Freshly thawed liver tissue (50 mg) was homogenised in 80 μL of normal saline and 2.63 μg/mL C11:0 as internal standard was added. Homogenates were kept on ice, then extracted using 1.5 mL of 2:1 chloroform-methanol (Sigma, UK) containing 0.01% tert-butylated hydroxytoluene (Sigma, UK). After centrifugation (200 x g for 10 min at 4 °C), the bottom layer containing the lipid was dried under nitrogen and retained for analysis.

Analysis of FA in fasting serum, and tissue was adapted from Pararasa et al. Pararasa et al. (2016). Methylation of NEFA was at 35 °C for 10 min using 200 μL toluene, 1.5 mL methanol and 0.3 mL HCl in methanol at 100 °C, with no significant hydrolysis of triglyceride under these conditions. FA methyl esters (FAMEs) extracts were separated by GC using an Omegawax 250 (fused silica bonded polyethylene phase) capillary column (30 m x 0.25 mm × 0.25 µm column). All GC-MS analyses were done at the Stable Isotope Laboratory, Leggett Building, Faculty of Health and Medical Sciences, University of Surrey, Guildford. Identification and quantification of FA peaks were determined against peak areas of a Supelco 37 FAME mix standard. In the GC-MS analyses, in addition to the internal standard (IS), a calibration curve was constructed for heptadecanoic acid (C17:0) against C11:0. A calibration plot of C17:0 compound was run by applying the ratio of the peak area of the FAME in the standards to the peak area of the IS against the ratio of the concentration of the FAME to the concentration of the IS. The concentration of FAME in the heptadecanoic acid solution was then determined using the area ratio and the calibration plot.

The composition of the FAs (μM) in the samples was then recalculated and used to determine percentage fatty acid composition.

## 4 Histology

Randomised collection of tissues were formalin-fixed then washed 3 times in PBS (Thermo-Fisher, UK), then embedded in paraffin. Liver Section (3 μm) were cut by microtome (Leica Biosystems, UK) and then mounted. Complete removal of paraffin was performed prior to staining of the section with haematoxylin and eosin. Section images were acquired using a DMR Leica microscope equipped with a High-End DP 72 Olympus digital camera (using ×60 lenses).

## 5 RNA isolation and qPCR

Total RNA was extracted from SPF mice livers using TRIzol™ (Thermo Scientific, UK) according to the manufacturer’s instructions. TURBO DNA-free™ Dnase treatment kit (Ambion Inc, USA) was used to remove any genomic DNA contamination. RNA sample concentration and quality were determined by A260/280 ratios, using a Nanodrop Lite Spectrophotometer (Thermo Scientific, UK).

RNA (2.0 μg) was converted to cDNA using NanoScript2 cDNA synthesis kit (Primerdesign, UK) then stored at − 20 °C until use.

RT-qPCR analysis was carried out using Stratagene (Thermo Scientific, UK) or Quant studio 7 Real-Time PCR system (Thermo Scientific, UK). RT-qPCR reactions for tissue samples were prepared using Precision Mastermix (Primer Design, UK) containing SYBR Green. Ct values were converted to relative expression values using the ΔCt method and reference gene stability was determined using the VBA applets for geNorm (Vandesompele et al., 2002) and NormFinder (Andersen et al., 2004). The data were normalised to two genes: *PGK1* and *TBP* that are stable in hepatic tissue. The primers for genes investigated, stearoyl co-A desaturase 1 (*SCD1*), fatty acid desaturase 2 (*FADS2*), ELOVL family member 6, elongation of long chain fatty acids (*ELOVL 6*), 2-hydroxyacyl-CoA lyase 1(*HACL1*), branched chain ketoacid dehydrogenase E1, α polypeptide (*BCKDHA*) and phytanoyl-CoA hydroxylase (*PCCA*), are reported in Table S1.

## 6 16 S ribosomal RNA gene sequencing

Faecal samples (~50 mg) were collected from the ileum at the end of the feeding study (*n* = 10 per group). Total genomic DNA was extracted using the MP Biomedicals DNA isolation kit (Fisher Scientific, UK) following the FastPrep system. The concentration and integrity of bacterial DNA were assessed using a Cubit (Thermo Scientific) and agarose gel electrophoresis, respectively. 16 S rRNA gene amplicon sequencing was performed by Novogene Ltd, Cambridge on the Illumina MiSeq platform using universal primers 341 F, 5′-CCTAYGGGRBGCASCAG-3′; and 806 R, 5′-GGACTACNNGGGTATCTAAT-3′ targeting the V3-V4 hypervariable regions.

## 7 Statistical analysis

For 16 S RNA analysis, raw data were merged and filtered to perform operational taxonomic unit (OTU) cluster and species annotation for the respective sequence of each OTU. The relative species, evenness and abundance distribution were analysed with alpha diversity and beta diversity. Downstream statistical analysis to explain the community construction differences between samples or among groups were performed via principal coordinate analyses (PcoA). Statistic methods such as *t*-test, MetaStat, LefSe, Anosim and MRPP were employed to test the significance of community composition and structure differences between groups.

Data were analysed using SPSS version 21 (Armonk, New York, USA). Descriptive analysis of biochemical data was performed for all variables with percentages and mean (standard deviation) as appropriate for normally distributed data, or median (range) for non-normally distributed data.

## 8 Results

**Fig. 1:**
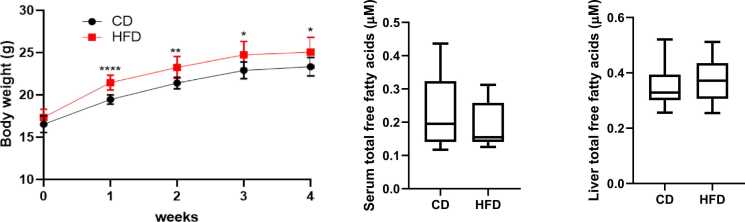
(a) High fat diet increases body weight in specific pathogen free (SPF) mice. Body weight gain was significant during the first 2 weeks of experimental feeding. ****p < 0.0001 and **p < 0.01 for high fat diet (HFD) relative to control chow diet (CD) and was maintained at 4 weeks; p < 0.05. SPF CD: n = 10; SPF HFD: n = 10. Fatty acid concentration in the (b) serum and from (c) livers of SPF mice after four weeks following high fat diet (HFD) relative to control chow diet (CD)**.** Values are given as median +/- 95% CI for n = 10 per group.

To explore the role of dietary fat intake on non-esterified OCSFA, we examined the effect of a high fat feeding diet (HFD) in SPF-housed C57B/6 mice. Initially, we characterised general biochemical parameters and observed over four weeks of HFD, that fasting serum glucose did not change (data not shown). However, from week one the SPF-housed mice had significantly higher body mass HFD that was sustained over four weeks (Fig. 1a). Despite HFD feeding for four weeks, total serum and liver fatty acid concentrations in SPF mice were not different from CD (Fig. 1b and c). Similarly, in standard husbandry over 4 and 12 weeks the total serum fatty acid conditions were not different between HFD and CD (Figure S1).

**Fig. 2:**
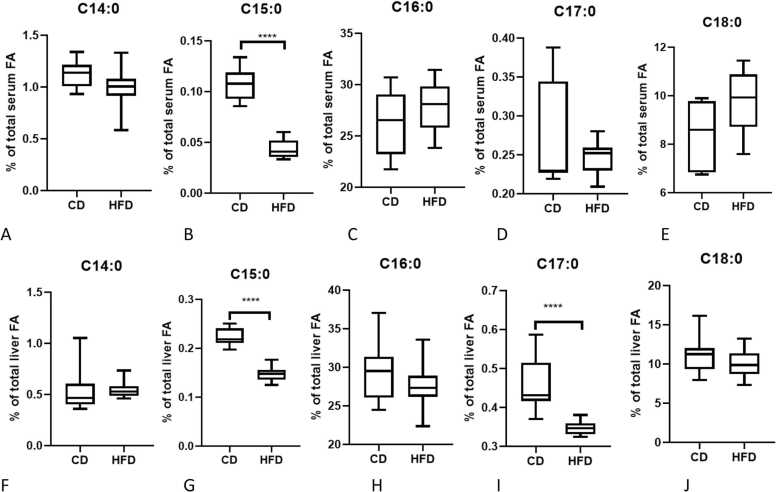
Effect of high fat intake on saturated FA proportion in the serum (A-E) and liver (F-J) in SPF mice. Fatty acids were measured in the serum of mice by GC-MS. N = 10 per group, ****p < 0.0001 (HFD vs CD).

To explore whether OCSFA levels were affected by HFD in mice, serum and liver non-esterified FA were measured. Total even-chain fatty acids (C14:0, C16:0 & C18:0) were not significantly different between HFD and CD fed groups in serum or liver tissues after 4 weeks (Fig. 2A,C,E,F,H,J). HFD mice had lower serum levels of C15:0 when compared to CD fed mice (Fig. 2B, p < 0.0001) but no significant difference in C17:0 was observed. In the livers from HFD mice, C15:0 (p < 0.05) and C17:0 (p < 0.05) levels were lower than the CD group (Fig. 2G&I). However, the absolute concentration of non-esterified OCSFA was higher in liver than in serum, indicative of local synthesis and/or storage of OCSFA in the liver. The variance observed in non-esterified FA concentration may reflect varying time from feeding to sampling between animals.

**Fig. 3:**
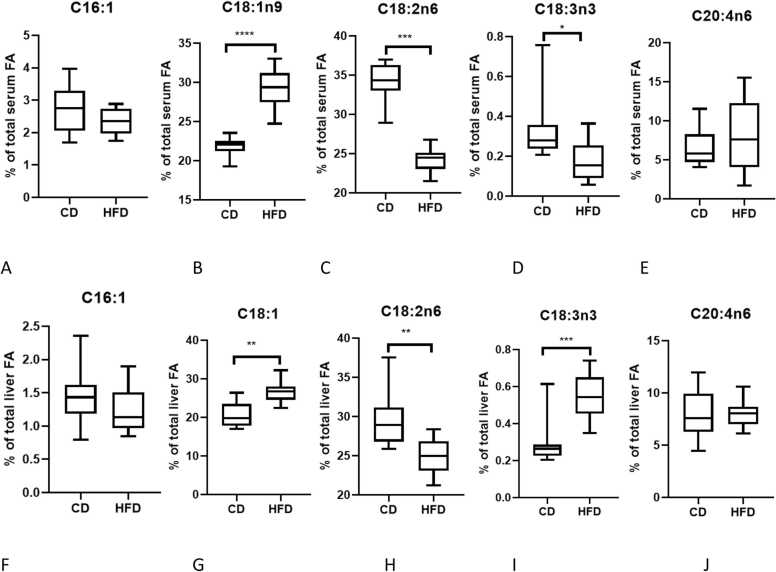
Effect of high fat intake on unsaturated FA proportion in the serum (A-E) and liver (F-J) in SPF mice, ****p < 0.0001; *** p < 0.001; ** p < 0.01 (HFD vs CD) n = 10 per group.

MUFA were significantly increased (p < 0.0001) in SPF-mice fed HFD compared to CD in both liver and serum, mainly due to increased C18:1n9, oleic acid (Fig. 3B&G). Total serum and liver PUFA were reduced after HFD. While arachidonic acid, C20:4n-6, was not different in either serum or liver between diets, linoleic acid levels, C18:2n6, were lower in HFD compared to CD fed mice (p < 0.001; Fig. 3C&H). In contrast, linolenic acid, C18:3n3, (p < 0.0001; Fig. 3D&I) was higher in liver but lower in serum after HFD, implying a localised change in metabolism following HFD.

**Fig. 4:**
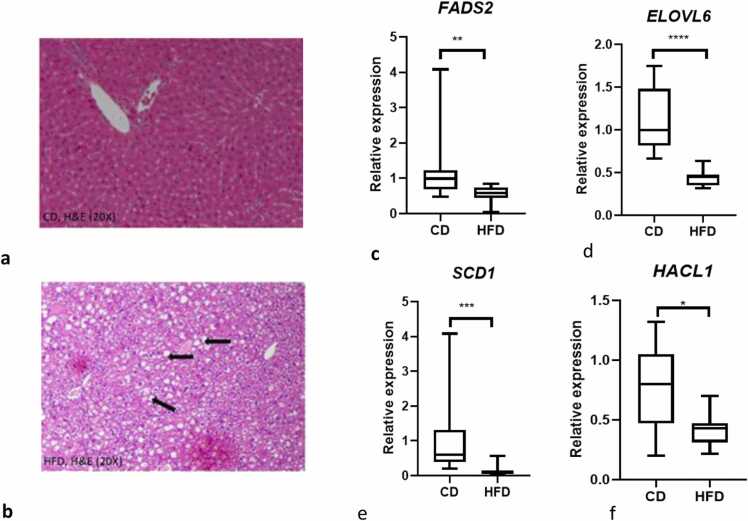
Hepatic steatosis (lipid vacuoles) in the livers of SPF mice on high fat diet but not control diet (a-b). Representative histology from chow diet group (CD) mouse and representative histology high fat diet (HFD) mouse liver. Liver sections stained with haematoxylin and eosin (H&E original magnification 20 ×). Effect of a 4-week HFD feeding on *ELOVL6, HACL1, SCD1, and FADS2* mRNA expression in SPF mice (c-f). Mean relative transcript expression of genes related to specific fatty acid changes in mouse liver. Values are given as means ± S.E.M for n = 10; *p < 0.05, *** p < 0.0001; ****p < 0.0001 CD vs HFD.

Consistent with the view that the liver accumulates fat after HFD, haematoxylin and eosin (H&E) staining from liver sections from CD fed mice showed normal architecture without any evidence of steatosis whereas HFD feeding induced histological steatosis in the liver (Fig. 4a&b).

To investigate whether the FA changes observed in the serum and liver of mice fed under SPF condition may be a consequence of altered hepatic gene expression, we analysed six genes involved in specific fatty acid synthesis and the inflammatory gene, TNFα. Expression of *SCD1*; (p < 0.001), *FADS2; (*p < 0.01), *ELOVL6*; (p < 0.0001), and 2-hydroxyacyl-CoA lyase 1, *HACL-1*, (p < 0.05) were significantly lower after a HFD compared to CD (Fig. 4c-f). Loss of HACL1, which degrades branched chain fatty acids in liver peroxisomes to yield C3:0, may contribute to lower circulating OCSFA. However, when we explored *TNFα*, *BCKDHA* (degrades branched chain amino acids to C3:0) and *PCCA* (activates C3:0 yielding propionyl CoA for synthesis of longer OCSFA) expression in livers from mice fed on HFD and CD there was no significant difference (data not shown).

An important source for liver-derived C3:0 is from the gut microbiota and is delivered directly via the hepatic portal vein, therefore we investigated the effect of HFD on the gut microbiome.

**Fig. 5:**
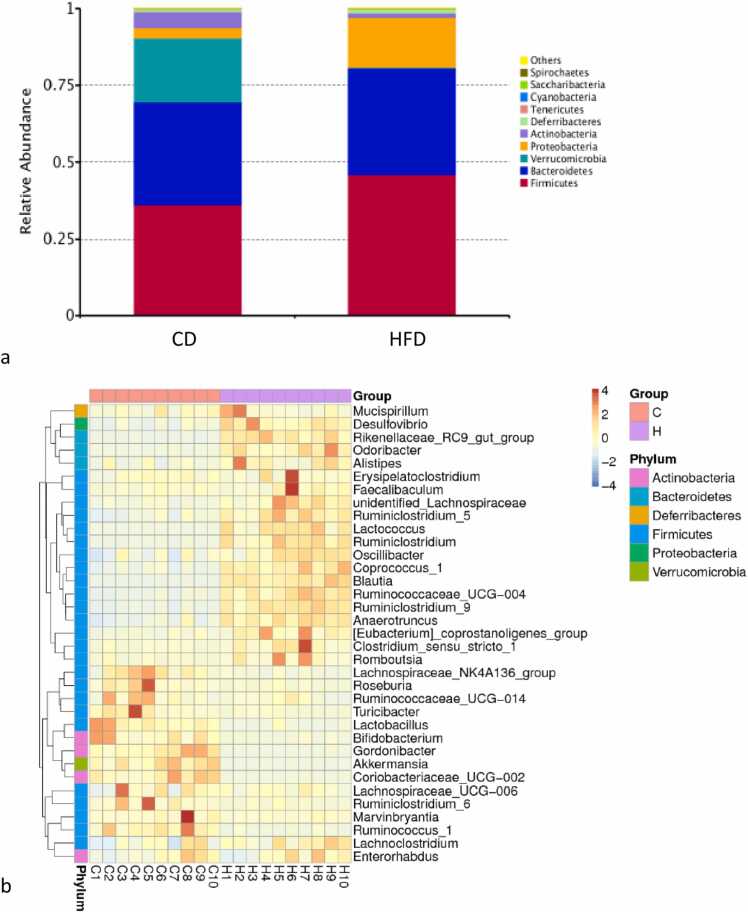
Specific dominant gut bacteria are affected by dietary fat. A: The composition of gut microbiota at phylum level; b: The heatmap of 35 differentially abundant genera between HFD and control treatment. Each column represented a sample from HFD and control treatment. (C: Controls (C1, C2, C3…); H: High fat diet (H1, H2, H3….).

After 4 weeks feeding with HFD, the proportion of Verrucomicrobia phyla was depleted compared to CD, however, Proteobacteria and Firmicutes phyla were increased in the HFD fed group (Fig. 5a). Specific genera of phyla Firmicutes that were increased by high fat diets included *Lactococcus* and *Clostridium*.

At the genus level, we observed 35 bacterial taxa that were differentially abundant between HFD and CD, as shown in the taxonomic abundance heatmap (Fig. 5b). Compared with the control group, 10 bacterial taxa were enriched in the HFD group, while 25 bacterial taxa were depleted in the HFD group. The HFD-enriched bacterial genera included *Clostridium, Eubacterium, Faecalibaculum, Erysipelatoclostridium, Desulfovibrio, Odoribacter, Alistipes, Romboustsia, Mucispirillum,* and unidentified members of Lachnospiraceae. Bacterial taxa that were depleted in the HFD group included *Roseburia*, *Lactobacillus*, *Akkermansia*, *Bifidobacterium*, which were significantly more abundant in CD (Fig. 5b).

## 9 Discussion

Epidemiological studies have identified that the prevalence of metabolic disease is inversely related to circulating OCSFA. Here we have explored this association in a short-term HFD feeding study of SPF mice. We observed that within four weeks, HFD-fed SPF mice became overweight without evidence of loss of glucose homeostasis, however, fatty droplets or vacuoles were evident in liver tissue. At the same time, liver C17:0 concentration, and liver and serum concentrations of C15:0 were lower than in CD-fed mice. The HFD diet contained higher OCSFA than the CD, hence dietary OCSFA intake does not explain the lower OCSFA concentration in serum after HFD.

Studies in vitro have shown that de-novo lipogenesis of OCSFA occurs in pre-adipocytes in the early stages of differentiation and in hepatocytes (Roberts et al., 2009). Altered metabolism in these tissues may contribute to changes in circulating OCSFA without changes in glucose homeostasis. Further suggestion that hepatic lipogenesis is affected by short term HFD feeding was obtained from the relative transcript expression of genes related to specific fatty acid metabolism in mouse liver. The lower expression of fatty acid desaturating enzymes observed here may predispose towards an increase in saturation of fatty acids produced in the biosynthetic pathway. Similarly, lower expression of *ELOVL6* observed here after a HFD may reduce chain length extension during fatty acid biosynthesis; *ELOVL6* has been shown by others to catalyse the elongation of OCSFA in human cells (Wang et al., 2019). We showed previously that lower abundance of long chain saturated fatty acids was associated with inflammation and ageing (Pararasa et al., 2016).

Another potential source of OCSFA is from the action of peroxisomal 2-OH acyl CoA lyase that cleaves and removes one carbon during fatty acid α-oxidation (Ampong et al., 2020). Liver expression of this gene was lower in HFD compared to CD-fed animals and may contribute to lower C17:0 and other have shown that 2-OH acyl CoA lyase is a major contributor to determining C17:0 levels.

Dysregulation of the gut microbiome has been linked to the pathogenesis of metabolic diseases, including fatty liver and T2DM (Campo et al., 2019). In addition, lower bacterial diversity is observed in people with inflammatory diseases, including psoriatic arthritis, T1DM, atopic eczema, coeliac disease, obesity, T2DM, and arterial stiffness (Valdes et al., 2018). When we investigated whether HFD affected the microbiome, we did not see any statistically significant change in microbiota diversity, however, phyla and genus abundances were affected. Increased Firmicutes and Proteobacteria phyla were observed after HFD whereas Verrucomicrobia, Actinobacteria, Saccharibacteria, Spirochaetes were decreased within the gut microbiota compared to CD-fed animals. Specific genera of phyla Firmicutes that were increased by high fat diets included C4:0-producing *Lactococcus* and *Clostridium*. Jung et al. have similarly reported an increase in *Lactococcus* and *Clostridium* genera following a high fat diet and was associated with glucose intolerance (Jung et al., 2016). The abundance of these genera was lower and glucose homeostasis was restored after resveratrol treatment. Others have also shown that a Western-type of diet in animals alters the microbiota, resulting in increased Firmicutes and decreased Bacteriodetes (Singh et al., 2017).

In our study, we observed that within the Verrucomicrobia phylum, C3:0-producing *Akkermansia spp*. were decreased; others have shown that these species maintain gut barrier function and are positively associated with improvement of NAFLD and T2DM (Everard et al., 2013). Furthermore, daily administration of *Akkermansia muciniphila* counteracted the deleterious metabolic effects of HFD in mice (Cani et al., 2012). In our study, *Bifidobacterium* and *Lactobacillus* were also reduced in HFD-fed SPF mice compared to CD feeding; both have anti-obesogenic effects in rodents (Li et al., 2016).

Short-term HFD feeding leads to a change in gut microbiota, including loss of taxa that produce odd, short chain fatty acids. It should be noted that OCSFA are biomarkers for dietary fat intake, since they are produced during α-oxidation in ruminants (Jenkins et al., 2015), however, the animals in this study were post-weaning.

A strength of this study is that it provides novel insight into the loss of non-esterified OCSFA after HFD. This was associated with changes in abundance of C3:0-producing gut bacteria and altered hepatic lipid metabolism. Further studies are required to identify any beneficial effect of C15:0, and of C3:0-producing gut bacteria to counter risk for metabolic diseases.

We thank Dr IHK Dias I, Dr D Burton, (School of Life and Health Sciences, Aston University) and Dr B Fielding (University of Surrey) for helpful discussions. We thank Dr Carla Moller-Levett and Dr N Jackson (University of Surrey) for advice on metagenomic analysis and on MS analysis respectively.

## Funding

HRG and DG acknowledge support from the 10.13039/501100000268BBSRC BB/M028100/2 and the 10.13039/100001642Glenn Foundation. OJI acknowledges support from Aston University for a postgraduate scholarship. IA acknowledges the Commonwealth PhD scholarship, GHCS-2016–146 Commonwealth Scholarships Commission, UK, 2016–2019. DG acknowledges support from the 10.13039/501100001809National Natural Science Foundation of China (NFSC) (Grant No. 81873665).

## CRediT authorship contribution statement

**Isaac Ampong**: Data curation, Formal analysis, Funding acquisition, Writing − original draft. **O. John Ikwuobe**: Methodology, Supervision, Writing − review & editing. **James EP Brown**: Methodology, Writing − review & editing. **Clifford J. Bailey**: Conceptualization, Supervision, Writing − review & editing. **Dan Gao**: Conceptualization, Supervision, Writing − review & editing. **Jorge Gutierrez-Merino**: Conceptualization, Supervision, Writing − review & editing. **Helen R. Griffiths**: Conceptualization, Funding acquisition, Supervision, Writing − original draft, Writing − review & editing.

## Declaration of interest

The authors declare that there is no declaration of interest associated with this manuscript.
