# Supplementary figures and images for: Odd chain fatty acid metabolism in mice after a high fat diet
Source: Int J Biochem Cell Biol. 2022 Feb;143:106135. doi: 10.1016/j.biocel.2021.106135 (PMC8811477; doi:10.1016/j.biocel.2021.106135)

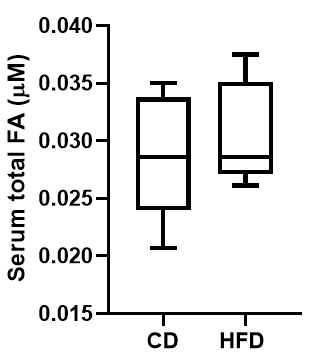

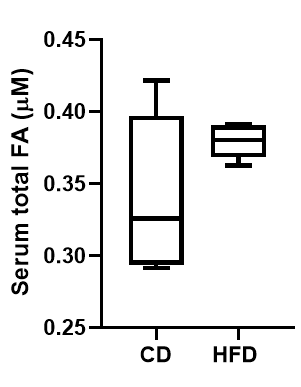


After 4 weeks After 12 weeks

Supplement: Supplementary file 1 — Supplementary material [file mmc1.docx]
