# Supplementary material for: Odd chain fatty acid metabolism in mice after a high fat diet
Source: Int J Biochem Cell Biol. 2022 Feb;143:106135. doi: 10.1016/j.biocel.2021.106135 (PMC8811477; doi:10.1016/j.biocel.2021.106135)
Supplement: Supplementary file 2 — Supplementary material [file mmc2.docx]

Supplementary Table 1. Primer sequences for qPCR in mouse tissue

| **Gene name** | **Forward sequence 5’-3’** | **Reverse sequence 5’-3’** |
| --- | --- | --- |
| *ELOVL 6* | cagggaggaagggctatgggcag | cgaacagggagggaggcgaaca |
| *PGK1* | tacctgctggctggatggaagacc | cacagcctcggcatatttct |
| *SCD1* | ttccctcctgcaagctctac | cagagcgctggtcatgtagt |
| *FADS2* | attcgggagaagatgctacg | aagaacttgcccacgaagtc |
| *PCCA* | acccactcaggcacaagcaaga | tttctctgccatctcgacaacttcc |
| *HACL-1* | agaactgccttcctcgccacagg | caccttccacacagatgacccgct |
| *TNFα* | tgacccctttactctgacccct | ggaccctgagccataatcccc |
